# Health-related quality of life in children and adolescents with celiac disease: survey of a population from central Italy

**DOI:** 10.1186/1477-7525-11-204

**Published:** 2013-12-05

**Authors:** Emma Altobelli, Riccardo Paduano, Teresa Gentile, Claudia Caloisi, Ciro Marziliano, Stefano Necozione, Ferdinando di Orio

**Affiliations:** 1 Department of Life, Health and Environmental Sciences, University of L’Aquila, Piazzale Salvatore Tommasi 1, Coppito (Aq), L’Aquila 67100, Italy; 2 Department of Pediatrics, San Salvatore Hospital, L’Aquila, Italy

## Abstract

**Background:**

Celiac Disease (CD) is an increasingly common autoimmune disorder. It requires a strict lifelong adherence to a gluten-free diet (GFD) which can influence health-related quality of life (HRQOL). This study assesses HRQOL in children and adolescents with CD and explores how several demographic and clinical characteristics and GFD adherence affect their perceived health status.

**Methods:**

We recruited 140 consecutive children and adolescents with CD confirmed by small bowel biopsy. HRQOL was assessed using the SF-12 questionnaire plus some CD-specific questions exploring wellbeing and lifestyle. Patients, aged 10 to 18 years, were identified by pediatric gastroenterologists and guided in filling out the questionnaire by trained psychologists. Parametric or non-parametric tests were applied to analyze continuous variables and frequencies as appropriate.

**Results:**

The SF-12 mean mental component summary score (MCS12) was lower than in the general Italian population (p < 0.001), whereas differences in terms of physical health were not significant (p = 0.220). More than one third of those interviewed reported feeling angry “always” or “most of the time” about having to follow the GFD, and nearly 20% reported feeling different from others and misunderstood because of CD “always” or “most of the time”.

**Conclusions:**

Our findings highlight the need for health professionals to identify adolescents with major disease-related problems. The food industry should improve its range of gluten-free food products and public bodies and institutions should promote informative campaigns and help promote the overall quality of life of children and adolescents with CD.

## Background

Coeliac disease (CD) is an immune-mediated systemic disorder triggered by the ingestion of gluten and related prolamines in genetically susceptible individuals [1]. It has been demonstrated that CD is one of the most frequent chronic childhood disorders, with a prevalence of approximately 1% in Western countries [2,3].

The only treatment for CD is avoidance of gluten-containing food. Lifelong compliance with a gluten-free diet (GFD) is crucial for prevention of long-term complications, disappearance of medical symptoms, and full histological and serological remission [2,4]. However, strict GFD adherence has the potential to affect patients’ lifestyle hence quality of life (QoL).

Studies of the impact of CD and GFD on health-related quality of life (HRQOL) have yielded conflicting results: whereas according to some Swedish [5,6], Canadian [7] and US [8] studies average HRQOL in adult patients was comparable to that of the general population, investigations conducted in Italy [9], Germany [10] and Northern Ireland [11] found a decreased HRQOL. However, the different methods used to measure HRQOL in the various studies limit comparability [12]. Several factors associated with a reduced HRQOL have been described in CD patients [13]; they include gender (female) [14,15], poor GFD adherence [9,16], older age at diagnosis [17], delayed CD diagnosis [18] and absence of symptoms [19].

Finally, studies involving children and adolescents are few. Although most QoL studies have examined adult CD patients, the disease clearly affects children and adolescents’ HRQOL too [19]. A considerable portion of these patients struggle to accept GFD, particularly adolescents aged 12 to 17 years [17].

This study assesses HRQOL in children and adolescents with CD and explores how several demographic and clinical characteristics and GFD adherence affect their perceived health status.

## Methods

### Patients

Recruitment took place from January 1 2011 to March 31, 2012.

Participants were Caucasian children and adolescents aged 10–18 years residing in Abruzzo (central Italy). They had a diagnosis of CD confirmed by small bowel biopsy according to the protocol of the European Society of Pediatric Gastroenterology, Hepatology and Nutrition, where small bowel biopsy followed by favorable clinical and serological response to GFD is considered sufficient to confirm the diagnosis [1]. Consecutive patients identified by pediatric gastroenterologists in the hospitals of the four Abruzzo provinces (L’Aquila, Pescara, Teramo and Chieti) were contacted by telephone and offered participation in the study. Those suffering from severe or terminal illnesses that might prevent participation were excluded. Participants were divided into groups of 10–20 and guided in filling out the questionnaire by trained psychologists. Written, informed consent was obtained from those of age (18 years) and from parental figures of minors. Strict anonymity was maintained via de-identification of data. The collected data were stored on a password-protected database.

### Data collection

For data collection we adopted the “Canadian Coeliac Health Survey”—a questionnaire developed by the Canadian Coeliac Association and the Medical University of Ottawa used in the study by Rashid et al. [20]—modified and improved based on the results of a pilot study of a group of teenagers followed at San Salvatore pediatric hospital (L’Aquila). Their data were not included in the analysis.

HRQOL was assessed using the Italian language version of the “Questionnaire on the Health Status SF-12 [21]. This questionnaire is constructed to measure 8 different domains: four refer to the area of physical health (Physical Functioning, Role Limitation-Physical, Bodily Pain, General Health) and four to mental health (Role Limitation-Emotional, Vitality, Mental Health, Social Functioning). Two synthesized indexes are derived from these 8 domains: one related to physical health (PCS12), the other to mental health (MCS12). Both summary scores are standardized to have a mean of 50 and standard deviation of 10, higher scores indicating better health perception.

To illustrate the effect of CD on the HRQOL of adolescents we compared our results with Italian normative values from a study conducted by ISTAT on a sample of 61,434 subjects, 3,221 (5.2%) of whom were aged 14–17 years [21]. Our questionnaire also included a set of specific questions about the wellbeing and lifestyle of children and adolescents with CD and their families regarding personal issues, social activities and potential interventions they felt would improve the QoL of celiac patients.

The self-report questionnaire was divided into two sections. One was administered to children and adolescents with CD, with items regarding social and demographic data, clinical symptoms at presentation, previous misdiagnoses, adherence to diet, and QoL and ways to improve it. The other was administered to pediatric gastroenterologists and solicited data on clinical features, to validate and broaden the information reported by patients. Children aged 10–13 years were helped by parents only in completing the sections related to demographic information and clinical features.

### Statistical analysis

The following parameters were used to estimate sample size: sample error = 0.06, event occurrence proportion 0.5 (in the case of maximum variability), probability 1-alpha = 0.95. This yielded a sample size of 136.1.

Continuous variables, presented as mean ± standard deviation (SD), were compared using Student’s *t*-test. The Kolmogorov-Smirnov test was performed in advance to check the normality of variables. The *χ*2 test was used to estimate the association between the categorical variables under study. Wilcoxon’s test and the Kruskal-Wallis test were applied to interval and ordinal variables. Logistic regression analysis was applied to evaluate whether there was a relationship between the HRQOL reduction and the variables examined. A value of p < 0.05 was considered statistically significant. SAS software, version 9.1.3 (SAS Institute, Cary, NC, USA), was used for the statistical analyses.

## Results

### Patients

For this study, 140 individuals with biopsy-confirmed CD were interviewed. All those who were invited to participate agreed to do so. The mean age of patients was 14.2 years (± SD 2.5); the majority were female (n = 110, 78.6%). Participants were divided into two age groups: children, aged 10–13 years (n = 63, 45.0%) and adolescents, aged 14–18 years (n = 77, 55.0%). Their demographic and clinical characteristics are listed and compared in Table 1.

**Table 1:** Demographic and clinical features of the sample: comparison between age groups

**Features**	**All cases (%)**	**Age groups**		**p**
		**10-13 years (n = 63)**	**14-18 years (n = 77)**	
**Gender**				
Males	30 (21.4)	12 (19.1)	18 (23.4)	0.53
Females	110 (78.6)	51 (80.9)	59 (76.6)	
**Age at diagnosis**				
0-6 years	64 (45.7)	33 (52.4)	31 (40.3)	0.15
≥ 7 years	76 (54.3)	30 (47.6)	46 (59.7)	
Symptoms*				
Symptomatic	39 (27.9)	22 (37.9)	17 (22.7)	0.06
Asymptomatic	94 (67.1)	36 (62.1)	58 (77.3)	
**Compliance**				
Strict	122 (87.1)	60 (95.2)	62 (80.5)	0.02
A little/none	18 (12.9)	3 (4.8)	15 (19.5)	
**Difficulty in compliance**				
A lot	16 (11.4)	9 (14.3)	7 (9.1)	0.01
Somewhat	27 (19.3)	8 (12.7)	19 (24.7)	
A little	58 (41.4)	21 (33.3)	37 (48.0)	
None	39 (27.9)	25 (39.7)	14 (18.2)	
**Duration of disease**				
0-3.5 years	40 (28.6)	22 (34.9)	18 (23.4)	0.31
3.6-8 years	47 (33.6)	20 (31.8)	27 (35.1)	
8.1-18 years	53 (37.8)	21 (33.3)	32 (41.5)	
**Previous misdiagnoses**				
Yes	50 (35.7)	14 (22.2)	36 (46.7)	< 0.001
No	90 (64.3)	49 (77.8)	41 (53.3)	
Delay in making the diagnosis**				
0-12 months	81 (57.9)	33 (64.7)	48 (67.61)	0.74
≥ 13 months	41 (29.3)	18 (35.3)	23 (32.39)	

* = 7 data missing. ** = 18 data missing.

**Figure 1:**
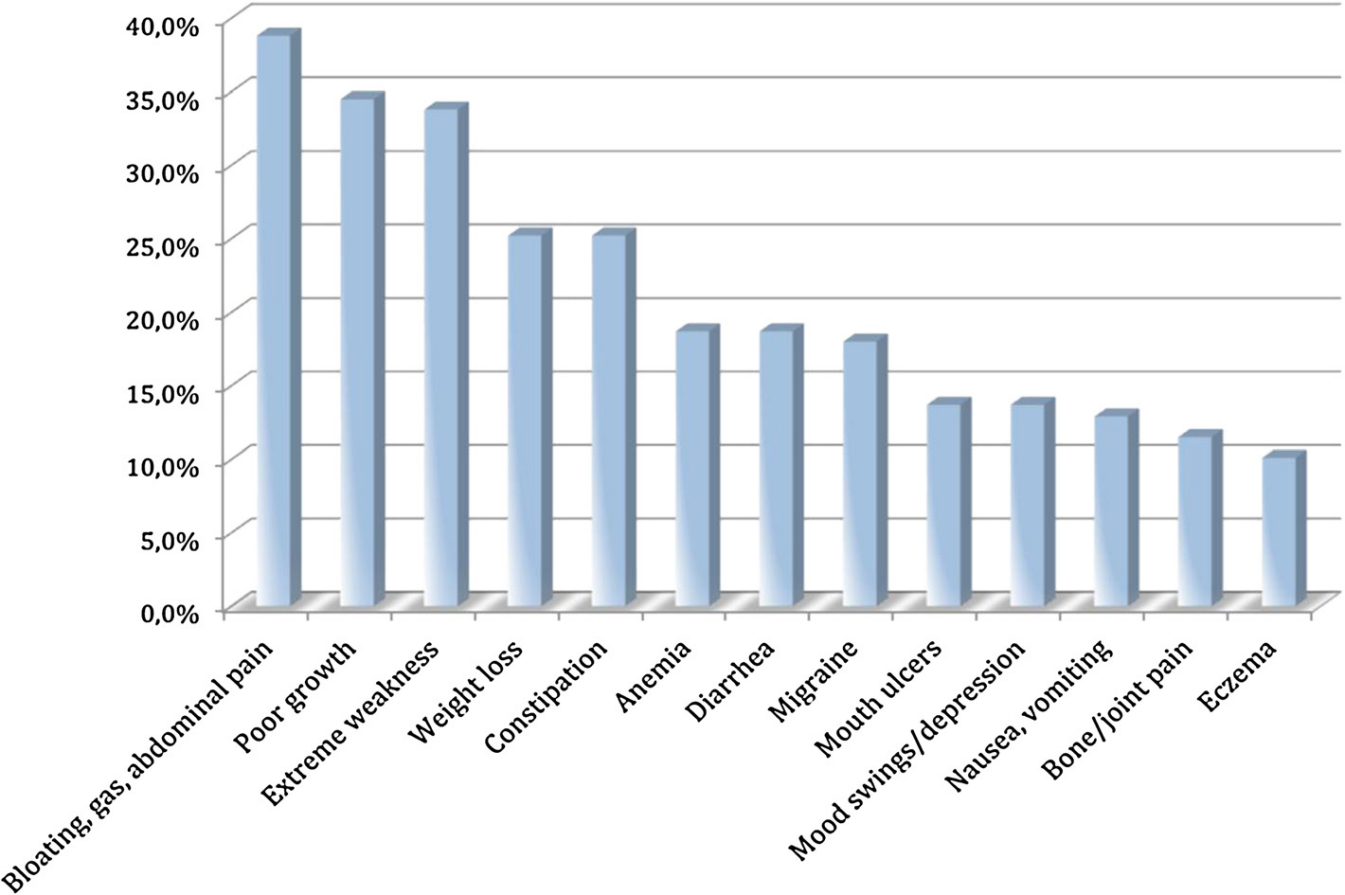
Clinical symptoms at CD presentation.

Mean age at diagnosis was 7.7 years (± SD 4.1) (males: 9.5 ± SD 3.6; females: 7.2 ± SD 4.2) with an average latency between symptom onset and CD diagnosis of 14.2 months (± SD 19.3). Fifty participants (35.7%) had received a previous diagnosis: anaemia and irritable bowel syndrome were the more common diagnoses (both 28.0%). Reported symptoms at presentation are listed in Figure 1.

In our sample, 39 participants (29.3%) were symptomatic at the time of diagnosis, and 94 (70.7%) were asymptomatic (data missing in 7 cases). A large proportion of symptomatic patients reported gastrointestinal symptoms after ingestion of gluten-containing food. Specifically, 26 (74.3%) patients reported diarrhea, 25 (71.4%) reported abdominal pain and 15 (42.9%) reported bloating and gas.

### Compliance with GFD

Almost all (97.9%) patients were advised to follow a GFD by their gastroenterologist. Strict adherence was reported by 122 (87.1%). Of these, 101 (73.2%) found a health benefit. Most found adherence to the diet to be “not difficult” (27.9%), or “a little difficult” (41.4%), while only 16 (11.4%) found adherence to be “extremely difficult” and 27 (19.3%) “quite difficult”. Ninety-eight (70.0%) subjects reported using the CD handbook to learn about the presence of gluten in food; 15.7% never used it and 14.3% did so “only sometimes”. Looking at the two age groups, children (aged 10–13 years) reported better compliance compared to older ones (p = 0.02) and less difficulty in adhering to the GFD (p = 0.01). Strict GFD compliance was reported by 95.2% of children, while 80.5% of adolescents did so. Additionally, 39.7% of children found GFD compliance to be “not difficult”, while only 18.2% of participants aged 14–18 years felt the same. No difference in the use of the CD handbook was found between the two groups.

### Quality of life

We assessed the QoL of adolescents with CD and the perception of how the disease influenced their social and personal life. Their mean PCS12 was 54.00 ± SD 5.00 and their mean MCS12 was 49.16 ± SD 10.61. The two summary scores were not normally distributed in each group (both p-values < 0.001).

To compare these data with Italian normative values, we looked only at the adolescent age group of the ISTAT study (14–17 years) [15]. No differences were observed in the physical health area (Table 2), whereas in the mental health area our sample showed a lower MCS12 score (p < 0.001). As shown in Table 2 the same difference was observed in both genders (males: p = 0.03; females: p < 0.001).

**Table 2:** Quality of Life: comparison between participants’ SF-12 score and Italian normative values

	**No. (%)**	**PCS12 mean ± SD**	**P**	**MCS12 mean ± SD**	**p**
**Age group 14–17 years**					
Italian average	3221 (5.2)	54.90 ± 4.69	0.22	54.05 ± 7.89	< 0.001
Sample	64 (45.7)	54.11 ± 5.07		48.45 ± 10.01	
**Males (14–17 years)**					
Italian average	1658 (51.5)	54.97 ± 4.58	0.40	55.17 ± 6.96	0.03
Sample	17 (26.6)	53.83 ± 5.43		50.93 ± 8.39	
**Females (14–17 years)**					
Italian average	1563 (48.5)	54.82 ± 4.80	0.41	52.87 ± 8.62	< 0.001
Sample	47 (73.4)	54.21 ± 4.99		47.55 ± 10.47	

We estimated the association between several variables and the scores of the sample on the physical and mental synthesized indexes of the SF-12. No differences were observed between genders, age groups, duration of disease categories, age at diagnosis, diagnostic difficulties (previous misdiagnoses and delays in making the diagnosis) or symptomatology at the time of diagnosis (data not shown). Compliance did not influence SF-12 results either. With regard to the perceived degree of difficulty in GFD compliance, those subjects who reported more difficulties had a lower MCS12 score, but the p-value was not significant (p = 0.08, data not shown).

The results of the binary logistic regression analysis are reported in Table 3. The independent variables analyzed did not significantly affect PCS12 or MCS12.

**Table 3:** Logistic regression analysis of factors predicting a reduced health-related quality of life as measured by the SF-12, in children and adolescents with CD

**Synthesized indexes**	**Independent variables**	**Odds ratio**	**95% CI**	**β**	**p**
**PCS12**	**Gender**	0.79	0.26-2.41	0.23	0.68
	**Age groups**	1.03	0.41-2.55	0.03	0.96
	**Age at diagnosis**	1.05	0.42-2.63	0.05	0.92
	**Symptoms**				
	**Compliance**	1.04	0.29-3.78	0.04	0.95
	**Difficulty in compliance**	1.80	0.29-3.78	0.04	0.95
	**Duration of disease**				
	**Previous misdiagnoses**	1.14	0.45-2.86	0.13	0.78
	**Delay in making the diagnosis**				
**MCS12**	**Gender**	1.27	0.53-3.07	0.24	0.59
	**Age groups**	0.79	0.37-1.72	0.23	0.56
	**Age at diagnosis**	0.98	0.45-2.14	0.02	0.96
	**Symptoms**				
	**Compliance**	0.91	0.30-2.74	0.10	0.87
	**Difficulty in compliance**	1.55	0.68-3.55	0.44	0.30
	**Duration of disease**				
	**Previous misdiagnoses**	1.07	0.49-2.31	0.06	0.87
	**Delay in making the diagnosis**				

Most of the items selected to assess the general wellbeing and lifestyle of children and adolescents with CD are summarized in Table 4. When looking at the age groups, no differences were observed in avoiding restaurants or in travel. Conversely, 14.3% of children reported bringing gluten-free food while travelling only “some of the time” or “never”, while only 2.6% of the older ones did so (p = 0.01). Difficulty in finding gluten-free food in stores was reported more frequently by adolescents (p < 0.01), as was difficulty in finding high-quality food (p = 0.03). On the other hand, children felt left out of activities more often than their older counterparts (p = 0.03). Gender did not influence most of the items, but girls felt more embarrassed about bringing gluten-free food to parties (p = 0.01, data not shown) and felt left out of activities more often (p = 0.02, data not shown). Division of the sample into symptomatic and asymptomatic patients yielded no difference in most of the items, except that symptomatic children and adolescents felt that they were not invited out for meals because of CD more often than asymptomatic ones (p < 0.01, data not shown), and worried more about staying in hospital because of CD (p < 0.001). Conversely, asymptomatic children and adolescents felt more often that they could be healthy without following a GFD (p = 0.01, data not shown). The analysis of age at diagnosis groups (0–6 years, ≥ 7 years) revealed no differences in the above items (data not shown).

**Table 4:** Children and adolescents perception of social and personal impairment

	**All cases**	**Age groups**		**p**
	**No. (%)**	**10-13 years**	**14-18 years**	
		**No. (%)**	**No. (%)**	
**Avoided restaurants**				
All the time	11 (7.9)	5 (7.9)	6 (7.8)	0.77
Most of the time	32 (22.9)	13 (20.6)	19 (24.7)	
Some of the time	50 (35.7)	21 (33.4)	29 (37.6)	
Never	47 (33.5)	24 (38.1)	23 (29.9)	
**Avoided traveling**				
All the time	4 (2.9)	0 (0.0)	4 (5.2)	0.14
Most of the time	7 (5.0)	4 (6.4)	3 (3.9)	
Some of the time	24 (17.1)	8 (12.7)	16 (20.8)	
Never	105 (75.0)	51 (80.9)	54 (70.1)	
**Brought gluten-free food while traveling**				
All the time	114 (81.4)	52 (82.5)	62 (80.5)	0.01
Most of the time	15 (10.7)	2 (3.2)	13 (16.9)	
Some of the time	9 (6.5)	7 (11.1)	2 (2.6)	
Never	2 (1.4)	2 (3.2)	0 (0.0)	
**Found it hard to find gluten-free food in groceries**				
All the time	16 (11.4)	6 (9.5)	10 (13.0)	< 0.01
Most of the time	19 (13.6)	9 (14.3)	10 (13.0)	
Some of the time	66 (47.1)	21 (33.4)	45 (58.4)	
Never	39 (27.9)	27 (42.8)	12 (15.6)	
**Found it hard to find good quality gluten-free food**				
All the time	15 (10.7)	4 (6.3)	11 (14.3)	0.03
Most of the time	21 (15.0)	9 (14.3)	12 (15.6)	
Some of the time	61 (43.6)	23 (36.5)	38 (49.3)	
Never	43 (30.7)	27 (42.9)	16 (21.8)	
**Found it difficult to determine if foods were gluten-free from labels**				
All the time	6 (4.3)	4 (6.3)	2 (2.6)	0.10
Most of the time	28 (20.0)	11 (17.5)	17 (22.0)	
Some of the time	43 (30.7)	14 (22.2)	29 (37.7)	
Never	63 (45.0)	34 (54.0)	29 (37.7)	
**Felt left out of activities at school or friend’s home**				
All the time	6 (4.3)	3 (4.8)	3 (3.9)	0.03
Most of the time	5 (3.6)	5 (7.9)	0 (0.0)	
Some of the time	30 (21.4)	9 (14.3)	21 (27.3)	
Never	99 (70.7)	46 (73.0)	53 (68.8)	
**Felt different from others cause of celiac disease**				
All the time	14 (10.0)	7 (11.1)	7 (9.1)	0.44
Most of the time	15 (10.7)	8 (12.7)	7 (9.1)	
Some of the time	47 (33.6)	24 (38.1)	23 (29.9)	
Never	64 (45.7)	24 (38.1)	40 (51.9)	
**Felt embarrassed to bring gluten-free food to parties**				
All the time	35 (25.0)	18 (28.6)	17 (22.1)	0.03
Most of the time	15 (10.7)	5 (7.9)	10 (12.9)	
Some of the time	34 (24.3)	9 (14.3)	25 (32.5)	
Never	56 (40.0)	31 (49.2)	25 (32.5)	
**Felt angry about the need to adhere to diet**				
All the time	29 (20.7)	10 (15.9)	19 (24.7)	0.14
Most of the time	21 (15.0)	9 (14.3)	12 (15.6)	
Some of the time	46 (32.9)	18 (28.6)	28 (36.4)	
Never	44 (31.4)	26 (42.2)	18 (22.4)	
**Felt that teachers and friends did not understand**				
All the time	6 (4.3)	2 (3.2)	4 (5.2)	0.65
Most of the time	21 (15.0)	9 (14.3)	12 (15.6)	
Some of the time	52 (37.1)	21 (33.3)	31 (40.2)	
Never	61 (43.6)	31 (49.2)	30 (39.0)	
**Thought of being a burden for someone because of celiac disease**				
All the time	10 (7.1)	5 (7.9)	5 (6.5)	0.42
Most of the time	16 (11.4)	6 (9.5)	10 (13.0)	
Some of the time	46 (32.9)	17 (27.0)	29 (36.7)	
Never	68 (48.6)	35 (55.6)	33 (42.8)	

### Keys to improve quality of life

Finally, participants were asked to choose from a list (not reported here) two items that could improve QoL for themselves and their families. The most frequent element chosen was “gluten-free choices in restaurants”, which was selected by 68.8% of participants; “gluten-free choices in supermarkets” was chosen by 36.0% and “informative campaigns in all social contexts” (e.g. schools, restaurants) by 36.6%; 32.9% of patients selected “better labelling”; 22.8% chose “earlier diagnosis”, and only 2.9% chose “better dietetic counseling”.

## Discussion

In this study we assessed the HRQOL and psychosocial wellbeing of 140 children and adolescents with CD from 140 families residing in Abruzzo (central Italy). To evaluate the CD features and treatment aspects that impact these outcomes most, we also assessed age at diagnosis, symptoms at presentation, diagnostic difficulties, disease duration, and compliance with the diet and related difficulties.

Mean age at diagnosis (7.7 years) was higher than that found by Rashid et al. (4.8 years) [20], but consistent with trends reported by Walker-Smith and March [22], highlighting an increasing age at presentation of children with CD throughout Europe. Our findings highlight that children present with a variety of signs and symptoms. This confirms the need for extending the indications for disease screening, because narrow thresholds may cause cases to be missed [4,22]. Although it is well known that CD is a common chronic childhood disorder, timely diagnosis is still lagging behind and the average interval between symptom onset and diagnosis remains long (14.2 months). Furthermore, one third of our sample reported previous incorrect diagnoses. Moreover, diagnosis by clinical signs only is impossible in many cases, because a large portion of CD patients are asymptomatic. It has been estimated that only 1 in 3 to 1 in 7 patients with CD are symptomatic [23,24]; the proportion of symptomatic patients in our study is consistent with the literature (29.3%).

In our sample 87.1% of participants reported “strict adherence” to GFD. Compliance was less “strict” than that reported by Rashid et al. (95%), but higher than the one found in a study conducted in Austria in 2008 [17] and in other European studies [14,25]. This could be due to the mean age of our sample (14.2 years), which is higher than that of the sample investigated by Rashid et al. (9 years), but similar to that of the Austrian (14.8 years) and the other European studies, where mean ranged from 14 years to “young adults”. In this regard, a Swedish study reported lower compliance among adolescents aged 15–17 years than among younger patients (12–14 years) [25]; our findings are in line with these data.

HRQOL has attracted considerable scientific interest in recent years. QoL is described as a perception of health and wellbeing impacting all aspects of our lives.

Numerous studies have investigated the influence of GFD on the HRQOL of the coeliac population [14,15,26,27] and have recognized that eating does not merely meet physiological needs: food has a symbolic value both in cultural terms and in the intra-psychic and relational aspects associated with it.

In general a decreased HRQOL is observed, especially in the social domain, but many questions remain concerning the impact of GFD on children and adolescents [28]. Our findings show impairment in both the mental and social domain of HRQOL. The mean mental health summary score (MCS12) of the SF-12 questionnaire was lower than that of the age- and sex-matched Italian population. Nevertheless, CD and CD treatment do not seem to affect physical health, consistent with previous findings showing that GFD usually results in an overall improvement in HRQOL [14,15].

Unlike other studies conducted in adults and children [14,15], which reported poorer HRQOL in females, the present survey found an equal burden of CD and the diet. This is in line with a recent investigation using the SF-36, from which the SF-12 has been obtained, conducted in Germany [13]; in contrast the other studies mentioned above used different types of questionnaires to investigate HRQOL.

Whereas Nörstrom et al. [18] found that delayed diagnosis is related to a decreased HRQOL in adults, our sample showed no difference in the physical, mental or social HRQOL domains. This may be due to the significantly lower average delay found in our study compared with that of Nörstrom et al. (14.2 months vs 9.7 years).

Late diagnosis did not entail significant differences in HRQOL in our sample. Such findings contrast with those reported by Wagner et al. [17]; this may however be ascribed to the different tools used to measure HRQOL, since they used the ILC (Inventory of Life Quality in Children and Adolescents), which found significant differences between patients diagnosed early and late, and the BFW (Berner Subjective Well-being Inventory) which found no differences between the groups.

Furthermore, diagnosing asymptomatic individuals with CD does not necessarily result in improved HRQOL. Although many studies have reported deteriorated HRQOL in asymptomatic CD patients diagnosed by screening [19,29,30], we found only minor differences between symptomatic and asymptomatic children and adolescents in personal issues and social activities, and no differences in SF-12 scores, in line with a recent study [31] that did not find a reduced HRQOL in asymptomatic adolescents detected through screening.

Poor GFD compliance, a factor that is associated with a reduced HRQOL in CD patients [9,16], did not relate with a decreased HRQOL in our sample, either.

Even though most of the children and adolescents interviewed adjusted well to GFD, more than one third felt angry “always” or “most of the time” about the need to follow the dietary regimen, and the same proportion felt embarrassed bringing gluten-free food to friends’ parties. Moreover, more than 20% of those interviewed reported feeling different from others because of CD “always” or “most of the time”, and 19.5% felt misunderstood. Only one third reported not avoiding restaurants ever, but this proportion rose to three quarters when travel was involved, with 81.4% writing that they always brought gluten-free food on journeys. Other difficulties reported included availability of gluten-free food in grocery stores (25% had difficulties “always” or “most of the time”), and determining from the label whether food was gluten-free (only 45% never succeeded). Significant differences between the age groups (10-13/14-18 years) were observed in finding gluten-free food in groceries: the older ones reported more difficulties. This may be explained by the fact that they are more involved in buying their own food, while younger patients rely on parents. Gender did not significantly influence social activities, except that girls felt left out of activities more often and felt more embarrassed about bringing gluten-free food to parties.

Overall, our findings are consistent with those of Rashid et al. [20] and with more recent studies [27,28] which found that the celiac adult population adhering to GFD reports a negative impact on HRQOL, especially in the social domain.

Finally, as regards potential interventions that could improve the QoL of celiac patients, the majority of those interviewed chose gluten-free choices in restaurants, and approximately one third chose informative campaigns in all social contexts and better labelling.

## Conclusions

Compliance with GFD results in an improvement in terms of the physical aspects of HRQOL. However, considering only this area fails to reveal the true extent of how gluten-free living impacts on HRQOL. Our study highlights that CD and its treatment result in a decreased HRQOL both in the mental and social domains.

Increased awareness of CD as a common worldwide public health problem [32] is needed and greater support and education should be given to CD patients to help them cope with the disease and its management.

In addition, as stated by children and adolescents with CD, the food industry should provide a wider choice of gluten-free food products, so as to make it more available to restaurants and groceries, as well as improve food labelling. Finally, public bodies and institutions should promote CD awareness campaigns and help promote the overall QoL of children and adolescents with CD.

## Competing interests

The authors declare that they have no competing interests.

## Authors’ contributions

EA, the principal investigator, designed the study, performed statistical analyses and wrote the article. RP did literature search, created the database, archived data and participated to write the article. TG participated in the clinical interpretation of data. CC participated to data collection. CM participated in statistical analyses. SN contributed in the interpretation of results and FdO critically revised the manuscript. All authors read and approved the final version of the manuscript.
